# Internalization of the *Aspergillus nidulans* AstA Transporter into Mitochondria Depends on Growth Conditions, and Affects ATP Levels and Sulfite Oxidase Activity

**DOI:** 10.3390/ijms21207727

**Published:** 2020-10-19

**Authors:** Sebastian Piłsyk, Adam Mieczkowski, Maciej P. Golan, Agata Wawrzyniak, Joanna S. Kruszewska

**Affiliations:** 1 Institute of Biochemistry and Biophysics, Polish Academy of Sciences, Pawińskiego 5A str., 02-106 Warsaw, Poland; amiecz@ibb.waw.pl (A.M.); jsk@ibb.waw.pl (J.S.K.); 2 Department of Neuropathology, Institute of Psychiatry and Neurology, Sobieskiego 9 str., 02-957 Warsaw, Poland; mgolan@ipin.edu.pl; 3 Morphological Sciences Department, College for Medical Sciences of University of Rzeszów, Leszka Czarnego str. 4, 35-615 Rzeszów, Poland; awawrzyniak@ur.edu.pl

**Keywords:** *Aspergillus nidulans*, alternative sulfate transporter, AstA, mitochondria, respiratory chain, pathogenic fungi, Hsp70, porin, thiol, sulfite oxidase

## Abstract

The *astA* gene encoding an alternative sulfate transporter was originally cloned from the genome of the Japanese *Aspergillus nidulans* isolate as a suppressor of sulfate permease-deficient strains. Expression of the *astA* gene is under the control of the sulfur metabolite repression system. The encoded protein transports sulfate across the cell membrane. In this study we show that AstA, having orthologs in numerous pathogenic or endophytic fungi, has a second function and, depending on growth conditions, can be translocated into mitochondria. This effect is especially pronounced when an *astA*-overexpressing strain grows on solid medium at 37 °C. AstA is also recruited to the mitochondria in the presence of mitochondria-affecting compounds such as menadione or antimycin A, which are also detrimental to the growth of the *astA*-overexpressing strain. Disruption of the Hsp70–Porin1 mitochondrial import system either by methylene blue, an Hsp70 inhibitor, or by deletion of the porin1-encoding gene abolishes AstA translocation into the mitochondria. Furthermore, we observed altered ATP levels and sulfite oxidase activity in the *astA*-overexpressing strain in a manner dependent on sulfur sources. The presented data indicate that AstA is also involved in the mitochondrial sulfur metabolism in some fungi, and thereby indirectly manages redox potential and energy state.

## 1. Introduction

To date, two different sulfate transporters were characterized in *Aspergillus nidulans*: main sulfate permease SB, the protein orthologous to sulfate permeases present in other fungi, and less known transporter AstA, found only in some fungal species [1,2]. The *astA* gene was cloned from a genomic library of Japanese *A. nidulans* strain IAM2006 as a suppressor complementing sulfate permease-deficient *sB* mutants [1]. Surprisingly, when comparing two strains of *A. nidulans* used in our previous studies, *astA* was functional only in Japanese strain IAM2006, while in Glasgow-derived referential strains, *astA* appeared to be nonfunctional and existed as a pseudogene [1,3]. The *A. nidulans* strains originating from Glasgow therefore contain only a single sulfate transporter, SB, of the sulfate permease (SulP) family. The AstA protein represents a weakly described type of sulfate transporters. It belongs to a broad and poorly characterized family of allantoate permeases, Dal5, from the major facilitator superfamily (MFS) [1]. Similar to *sB*, the expression of the *astA* gene is tightly regulated by sulfur metabolite repression (SMR), and activated under sulfur limitation conditions [1]. It was also shown that AstA can transport both sulfite and choline-*O*-sulfate ester [4]. Orthologs of *astA* occur frequently in evolutionary distant fungi belonging to the *Dikarya* phylum, often endophytes or pathogens of plants or animals. Japanese *A. nidulans* strain IAM2006, bearing the functional *astA* gene, was found primarily as a parasitic contamination of unshelled rice, causing significant damage in stored grains [5]. In contrast, the Glasgow reference strain of *A. nidulans*, carrying the truncated *astA* pseudogene, was isolated from soil as a harmless saprophyte [6]. An *astA* ortholog identified from potato pathogen *Fusarium sambucinum* was found to be derepressed during tuber infection, and the AstA protein was shown to be involved in efficient sulfate uptake [7].

**Figure 1:**
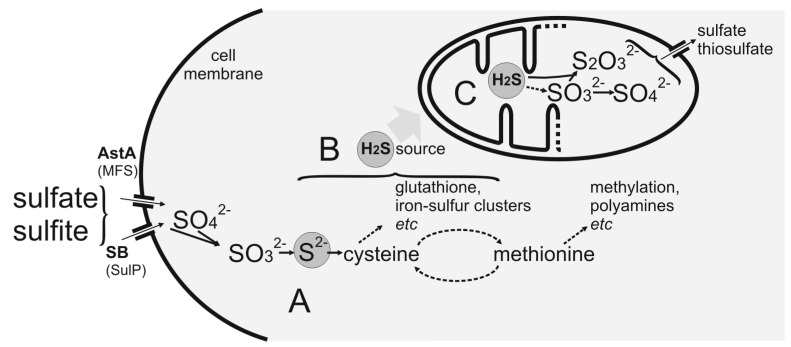
Sulfur metabolism in fungal cell. (**A**) Sulfur-containing amino acids; (**B**) sulfide sources; (**C**) mitochondrial catabolism.

All inorganic oxidized sulfur sources taken up by SB or AstA such as sulfate or sulfite are gradually reduced in the cell of prototrophic fungi, and the sulfide anion is incorporated into sulfur-containing amino acids cysteine and methionine (Figure 1A). Sulfur amino acids are required for protein synthesis, and they also participate in numerous biochemical pathways such as methylation processes, polyamine synthesis (methionine), or mitochondrial iron–sulfur-cluster (cysteine) synthesis. The side effect of these processes is the release of toxic hydrogen sulfide by enzymes handling the labile S^2–^ ion (Figure 1B), which is metabolized and detoxified to harmless sulfate in mitochondria (Figure 1C) [8].

Studies in human cells showed that the steady-state concentration of sulfide is a product of enzymatic activity in trans-sulfuration (cystathionine β-synthase and γ-cystathionase) and cysteine catabolic (mercaptopyruvate sulfurtransferase) pathways [9,10]. According to the current knowledge, the mechanism of sulfide detoxification in mitochondria follows through the pathway composed of sulfide:quinone reductase (SQR), sulfide dioxygenase (SDO), and rhodanese. The resulting sulfite is lastly oxidized to harmless sulfate by sulfite oxidase (SOX), localized in the intermembrane space of the mitochondria. It was also shown that sulfide might function as a mediator in oxygen sensing through cellular switching between low steady-state intracellular H_2_S level and higher concentrations that arise under hypoxic conditions [8]. Under these conditions, the activities of SQR and sulfur dioxygenase are inhibited, which leads to increased H_2_S levels, inhibition of cytochrome *c* oxidase, ATP insufficiency, and consequent triggering of the hypoxic response [11].

Contrary to ATP-dependent SulP sulfate permease, which is present in all fungi, sulfate transport by the ATP-independent AstA seems to be an evolutionarily beneficial trait [3]. It is especially crucial for endophytes or pathogens, where their hydrolytic activity of host tissue often occurs under low-oxygen conditions and subsequent limited ATP synthesis.

Here, we demonstrate that the *A. nidulans* AstA sulfate transporter is also internalized into the mitochondria under altered culture conditions when the fungus grows on solid medium and at 37 °C. Depending on the available sulfur source, AstA modulates mitochondrial ATP level and sulfite oxidase activity. Moreover, known mitochondria-affecting compounds such as reactive oxygen species (ROS)-generating menadione or Complex III-inhibiting antimycin A also trigger AstA translocation into the mitochondria.

One of the mitochondrial import pathways leads through Porin, an oligomeric protein located in the mitochondrial outer membrane. Porin1, also known as the voltage-dependent anion channel (VDAC-1), participates in the mitochondrial import of multiple proteins and other low-molecular-weight compounds [12,13]. In humans, the Hsp90 chaperone was shown to primarily facilitate the import of proteins into the Porin1 channel with less Hsp70 involvement, whereas in fungi, Hsp70 is mostly responsible for this import [14]. Under certain stress conditions, such as elevated temperature, Hsp70 is activated by its oligophosphorylation [15,16]. Hsp70 also plays a crucial role upon biofilm formation by fungi, such as the pathogenic *Candida albicans* [17,18,19,20].

In this paper, we show that AstA translocation into the mitochondria is mediated by the Hsp70–Porin1 import system. Phenotypic tests suggest that AstA is involved in mitochondrial redox balance, sulfite efflux, and respiratory-chain function. Regarding the fact that the *astA* gene is found in some pathogenic or endophytic fungi, its function in respiratory-chain tuning may be considered as an evolutionary adaptation for low ATP consumption under microaerophilic conditions.

## 2. Results

### 2.1. Conservation of the astA Gene in Pathogenic or Endophytic Fungi of Dikarya Phylum

**Figure 2:**
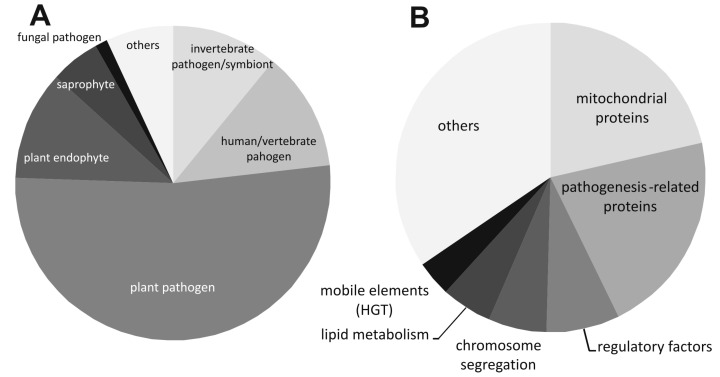
Pie charts of fungal lifestyles and proteins encoded by genes located near *astA*. (**A**) Lifestyle of *astA*-bearing fungi represented mostly by pathogens, symbionts, or endophytes. (**B**) Cellular processes involving proteins encoded by genes located in 5′ and 3′ adjacent regions of the *astA* loci in various fungi.

After screening the GenBank protein and genome database, numerous sequences orthologous to AstA were found, mostly in pathogenic or endophytic fungi colonizing plants, insects, or vertebrates, and, occasionally other fungi (Figure 2A, Supplementary Table S1). All of them belong to the *Dikarya* subkingdom, represented by the *Pezizomycotina* and *Basidiomycota* phyla. Interestingly, AstA is apparently absent from the *Saccharomycotina* subphylum. Among *Pezizomycotina astA* appears in the orders of *Orbiliales*, *Chaetothyriales*, *Phaeomoniellales*, *Verrucariales*, *Eurotiales*, *Helotiales*, *Glomerellales*, *Hypocreales*, *Microascales*, *Diaporthales*, *Magnaporthales*, *Ophiostomatales*, *Sordariales*, *Togniniales*, *Xylariales*, *Capnodiales*, *Myriangiales*, *Dothideales*, *Venturiales*, *Hysteriales*, and *Pleosporales*. Among basidiomycetous fungi, two subphyla (*Agaricomycotina* and *Pucciniomycotina*) carry *astA*, represented by the orders of *Holtermanniales*, *Filobasidiales*, *Tremellales*, *Trichosporonales*, *Agaricostilbales*, *Erythrobasidiales*, *Cystobasidiales*, *Microbotryales*, *Pucciniales*, and *Auriculariales*. While looking for potential pathogenic islands, including encompassing genes to the *astA* loci, we found that many vicinal genes located in 5′ or 3′ regions of *astA* are frequently involved in mitochondrial metabolism (over 21%: proteins involved in the Krebs cycle and its protection, such as chaperones, nitrite oxidase, and mitochondrial energy-recycling sarcosine oxidase), pathogenesis (over 21%: sodium channels, proteins with CFEM domain, glycohydrolases), chromosome segregation (metalophosphatase, F-BAR, Smc, SepB, HET-heterokaryon incompatibility), mobile elements (transposones, RNase H, RT polymerases, transposases), regulatory factors (C6 zinc finger transcription factors and C2H2, RING finger proteins), or lipid metabolism (mainly mitochondrial enzymes and the C6 transcription factor, orthologous to protein MANI_116542, involved in lipid biosynthesis) (Figure 2B).

### 2.2. Redox-Reactive Sulfur Sources or Altered Redox Conditions Are Detrimental for Growth of an astA-Overexpressing Strain

**Figure 3:**
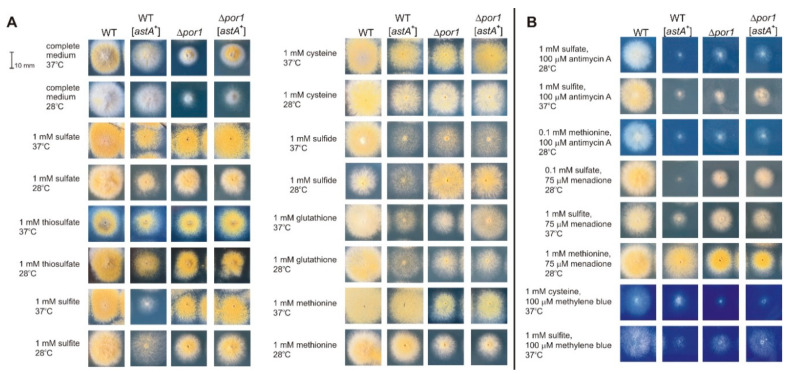
Phenotypic tests of the wild-type strain (WT), an *astA*-overexpressing strain (WT [*astA*^+^]), a porin1 mutant (Δ*por1*), and a porin1 mutant overexpressing *astA* gene (Δ*por1* [*astA*^+^]). Growth tested on solid complete (organic) or minimal (inorganic defined) medium supplemented with (**A**) different sulfur sources with several combinations of menadione, (**B**) antimycin A and methylene blue. Cultures carried out at 28 or 37 °C (as indicated in descriptions) for 24 h.

Commonly used temperatures for the cultivation of *A. nidulans* (28 or 37 °C) did not significantly affect strain morphology or growth rate (Figure 3A). Retarded growth of the *astA*-overexpressing strain was observed on media supplemented with inorganic sulfur sources or thioles (cysteine, glutathione), as compared to the wild-type strain. Furthermore, the ΔPor1 mutant and ΔPor1 *astA*-overexpressing strain showed retarded growth under these conditions, with exception of the medium supplemented with inorganic sulfide at 28 °C. However, mitochondria-affecting compounds such as the ROS-generating compound menadione or Complex III-inhibiting antimycin A were detrimental to the growth of the *astA*-overexpressing strain (Figure 3B). Their sensitivity depends on the available sulfur source, especially when the *astA*-overexpressing transformant uptakes redox-reactive sulfur compounds (sulfhydryl thioles such as cysteine or sulfite). Methylene blue, a known Hsp70 inhibitor [21], also affects the growth of the *astA*-overexpressing strain in the presence of reactive sulfur sources (Figure 3B).

### 2.3. Dual-Cell-Membrane and Mitochondrial Localization of AstA Depends on Culture Conditions and Is Achieved via N-Terminal Cytoplasmic Targeting Signal

**Figure 4:**
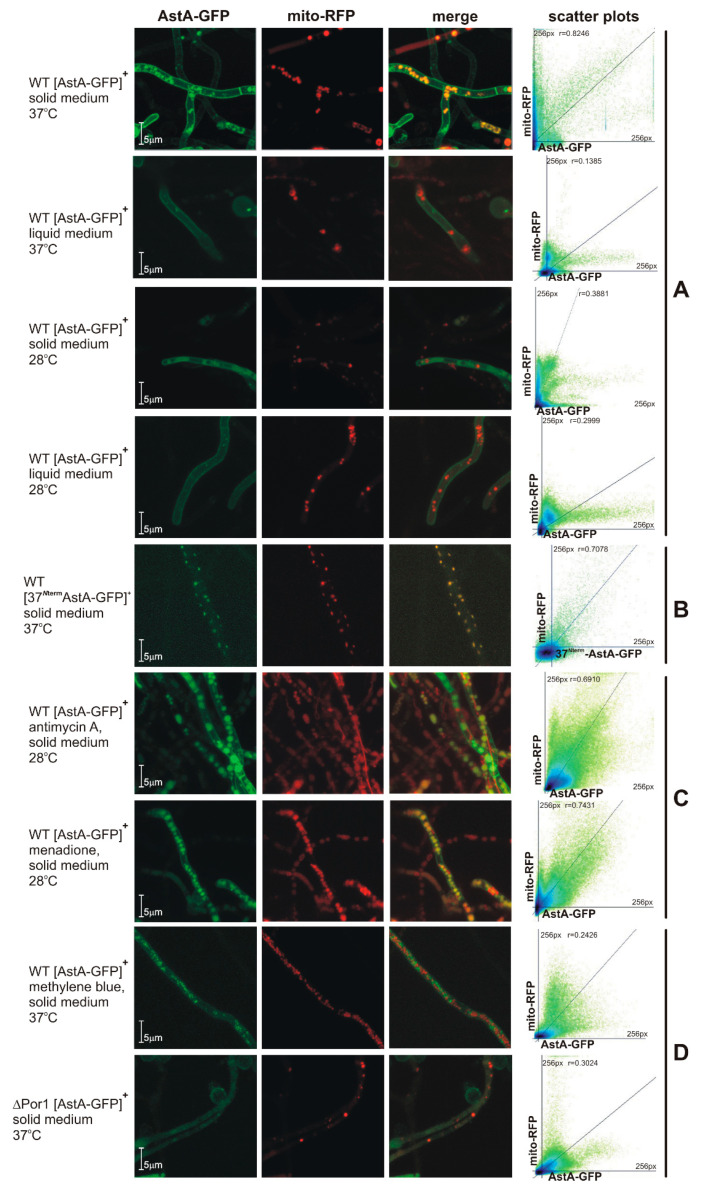
(**A**) Localization of AstA-GFP or 37^Nterm^AstA-GFP in *A. nidulans* strains under various growth conditions. Mitochondrial localization of AstA-GFP in hyphae grown on solid medium at 37 °C, or in cell membrane when same strain was grown in liquid minimal medium (MM) at 28 or 37 °C, or on solid medium at 28 °C (MM supplemented with 0.1 mM sulfate in all experiments). (**B**) Solely mitochondrial localization of hybrid 37^Nterm^AstA-GFP protein bearing 37 *N*-terminal amino acids of AstA. (**C**) Either reactive oxygen species (ROS)-generating and glutathione (GSH)-depleting drug menadione (75 μM) or Complex III-inhibiting antimycin A (100 μM) redirects AstA to the mitochondria at a lower temperature of 28 °C. (**D**) Hsp70-inhibiting compound methylene blue (100 μM) or mutation of porin1 prevents mitochondrial localization of AstA at 37 °C. (**right column**) Scatter plots and Pearson’s coefficient of two protein signals.

Growth of the *astA*-overexpressing strain was impaired in the presence of mitochondria-disturbing compounds (Figure 3B). In our previous studies, we showed that the cultivation of *A. nidulans astA*-GFP expressing strain in liquid minimal medium at 37 °C resulted in the predominant localization of GFP-tagged AstA in the cell membrane [3]. In this study, we found that, when the strain was cultivated on solid medium at 37 °C, the AstA-GFP signal was mainly translocated into the mitochondria (Figure 4A).

*In silico* prediction of the subcellular localization of AstA showed only cell-membrane targeting. Looking for a mitochondrial targeting signal, we created a hybrid construct by fusing the 5′ fragment of the *astA* gene (encoding *N*-terminal, cytosolic 37 amino acids) to the GFP-encoding reporter sequence (Figure 4B). The *A. nidulans* WT strain bearing this construct showed the WT phenotype in all growth conditions (data not shown); when it was cultivated at 37 °C on solid medium, the fluorescence of GFP was localized only in the mitochondria. Thereby, when the strain grows on solid medium at 37 °C, the *N*-terminal 37 amino acids of AstA are sufficient to recruit 37^Nterm^AstA-GFP to the mitochondria.

Since menadione and antimycin A significantly affect the growth of the *astA*-overexpressing strain (Figure 3B), it was of interest to check AstA-GFP localization in the presence of these compounds at 28 °C, when AstA-GFP would be expected to be localized in the cell membrane. The *A. nidulans* strain expressing AstA-GFP was cultivated at 28 °C, and both the ROS-inducing menadione and the complex III-inhibitor antimycin A triggered AstA-GFP translocation into the mitochondria under these conditions (Figure 4C), while the Complex I inhibitor, rotenone, did not (data not shown).

### 2.4. AstA Internalization into the Mitochondria via Hsp70–Porin1 Import System

Looking for the import system responsible for AstA translocation into the mitochondria, we first studied AstA-GFP localization in the presence of methylene blue, a known Hsp70 inhibitor [9], which also altered the growth of strains overexpressing *astA*. The lack of AstA-GFP in the mitochondria (Figure 4D) during cultivation in methylene blue-supplemented medium suggested that the Hsp70-Porin1 import system might be involved in this internalization. To confirm this, an *Anpor1::pyr-4* mutant was created by disrupting the AN4402 gene, which encodes a protein homologous to Porin1. The obtained Δ*por1* mutant showed slightly retarded growth on complete medium, although mitochondria-disturbing agents (menadione or antimycin A) or methylene blue affected its growth (Figure 3B). The lack of AstA-GFP in mitochondria of the Δ*por1* mutant confirmed that the Hsp70–Por1 system is involved in AstA internalization into the mitochondria (Figure 4D).

### 2.5. Altered ATP Levels and Sulfite Oxidase (SOX) Activity in astA-Overexpressing Strain Dependent on Sulfur Sources

**Figure 5:**
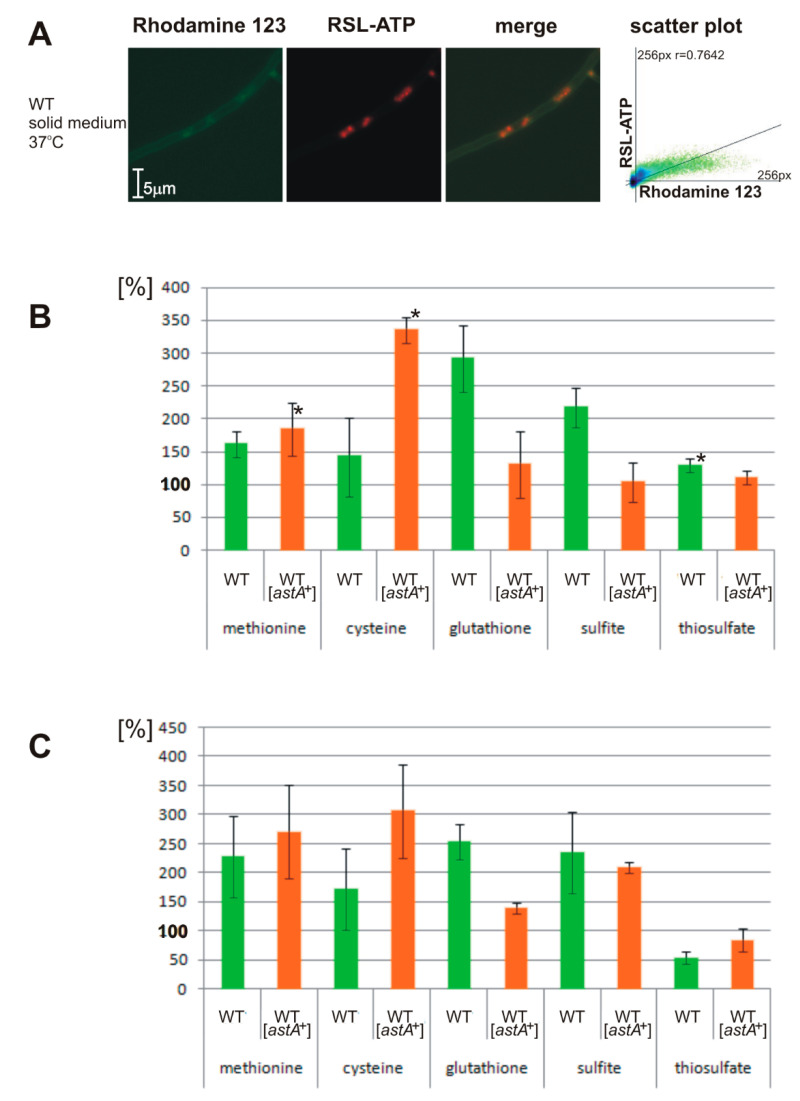
ATP levels and sulfite oxidase (SOX) activity in *astA*-overexpressing strain depend on sulfur sources. (**A**) Representative visualization of RSL-based detection of ATP, which is localized mostly in the mitochondria. (**right**) Scatter plot and Pearson’s coefficient of two fluorescent signals. (**B**) Relative ATP amount or (**C**) SOX activity ratio (as a percentage, assuming 100% of ATP amount/SOX activity in the wild-type strain grown on 1 mM sulfate, final oxidation product of sulfur compounds in the mitochondria) in crude extracts of the wild-type and *astA*-overexpressing strain, cultured on media supplemented with 1 mM: methionine, cysteine, glutathione, sulfite, or thiosulfate. Values correspond to mean of three independent experiments and SD. *, statistically significant results compared to control (1 mM sulfate) by Student’s *t*-test with *p*-value < 0.05.

Due to the mitochondrial targeting of AstA, it was interesting to study how its localization affects ATP levels and the activity of sulfite oxidase (SOX), the last enzyme of the mitochondrial sulfite oxidation pathway. To this aim, both the wild-type strain and the *astA*-overexpressing mutant were grown on solid medium, supplemented with various sulfur sources. Subsequently, the amount of ATP was estimated using a fluorescent ATP-specific sensor (Materials and Methods). Its ATP specificity was validated under a microscope to confirm its colocalization signal in the assayed cells with Rhodamine 123-labeled mitochondria (Figure 5A). Measurements of ATP amount in crude extracts revealed that the overexpression of *astA* leads to over twofold higher production of ATP when cysteine is used as a sulfur source compared to the wild-type strain. However, other sulfur compounds either decrease ATP levels or affect them insignificantly (methionine), as compared to the wild-type strain (Figure 5B). SOX activity is correlated with ATP levels (with the exception of thiosulfate), with elevated activity upon cysteine addition (Figure 5C).

## 3. Discussion

The *astA* gene is found in numerous fungi from the *Dikarya* subkingdom, representing pathogens or endophytes (Figure 2A), whose lifestyles require growth under microaerophilic conditions, elevated temperature, surface growth, or upon biofilm formation, for instance, when penetrating the host tissue. Looking for potential pathogenic islands encompassing the *astA* loci, we found that, in numerous fungi, adjacent genes are prevalently involved in mitochondrial metabolism or carbohydrate activity.

**Figure 6:**
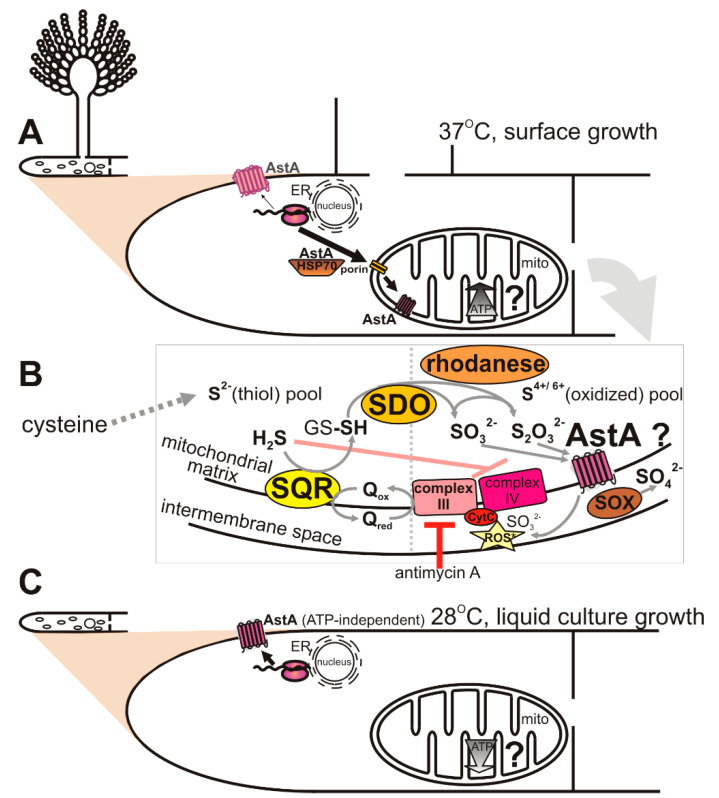
Proposed model of AstA function in fungi expressing the *astA* gene. (**A**). Hypothetical translocation pathway of AstA to the mitochondria under elevated-temperature and surface-growth conditions via Hsp70–Porin system. (**B**). Hypothetical function of AstA in the mitochondria upon conditions of 37 °C and solid culture growth. (**C**). Localization of AstA in the cell membrane at low temperature or under liquid-growth conditions.

In this paper, we showed that AstA is internalized into the mitochondria under certain stress conditions, such as vegetative surface growth on a solid medium, especially when combined with a temperature of 37 °C (Figure 6A). In routine laboratory practice with fungi, they are usually grown at 28 °C, although some species, such as *A. nidulans*, are commonly cultivated at 37 °C. This temperature is not considered to be stressful for *A. nidulans*, although it may trigger some heat-shock mechanisms, such as Hsp70 activation, and it caused AstA translocation into the mitochondria. Moreover, we found that the shift of AstA to the mitochondria was promoted by mitochondria-disturbing agents, such as ROS-generating and glutathione (GSH)-depleting menadione [22,23] or Complex III-inhibiting antimycin A [24,25]. The localization of AstA in the mitochondrial membrane was not obvious, since none of the available prediction software indicated that AstA targets mitochondria. Nevertheless, the *N*-terminal 37 amino acids from the AstA sequence were shown to serve as a cryptic mitochondrial leader signal, and remained sufficient for recognition by Hsp70, which recruited the hybrid 37^Nterm^AstA-GFP protein to the mitochondria.

Dual targeting was described for many mitochondrial proteins [26], and it may be achieved in a number of ways, for example, two (or more) transcription products from one locus, multiple translation products from one mRNA, an ambiguous targeting signal, two targeting signals, accessibility of targeting signals, or reverse translocation. A bimodal targeting signal was found in mammalian cytochrome P450 monooxygenases (CYPs), detected both in the endoplasmic reticulum and in the mitochondria [27], or for fumarase and aconitase, which share their occurrence between the cytosol and the mitochondria [28].

Therefore, it was interesting to find the system responsible for AstA translocation into the mitochondria and analyze its function there. Among known mitochondrial import systems, the porin channel is one of the main ones. In *A. nidulans,* deletion of the *por1* gene encoding Porin1 or inhibition of Hsp70 by methylene blue prevents AstA translocation into the mitochondria. These results led us to the conclusion that transport of AstA to the mitochondrial membranes is carried out by the Hsp70–Porin1 mitochondrial import system.

It was previously shown that recruitment of mitochondrial targeted proteins by the Hsp70 chaperone is controlled by ATP binding and hydrolysis, and involves two types of cochaperones [29]. In addition, activation of Hsp70 was triggered by stress conditions, e.g., elevated temperature [16,17], or upon biofilm formation in yeasts, such as *C. albicans* [17,18,19,20]. This agrees with our results, where the recruitment of AstA to the mitochondria was observed at 37 °C and during stress conditions induced by mitochondria-disturbing agents.

Many proteins are translocated to the mitochondria or other organelles by the Hsp70 system under certain growth conditions [12,13,14]. Since the frequently used technique of culturing fungi on glass slides for microscopic studies may lead to false conclusions, results need to be interpreted with caution.

Although the AstA function in the cellular membrane is well-established [1,3], the mechanism of AstA action in the mitochondria seems to be more complex, putatively involving respiratory-chain protection and participation in the last step of the sulfide-detoxification pathway. Regarding the fact that AstA is also able to transport sulfite and thiosulfate, this transporter may be responsible for their efflux out of the mitochondrial matrix, improving respiratory efficiency (Figure 6B).

Besides the assimilation pathway, sulfate and sulfite appear in the mitochondria upon detoxification of hydrogen sulfide, a toxic gas, which (like cyanide) shuts down cellular respiration by the inhibition of Complex IV, cytochrome *c* oxidase (Figure 6B) [30]. Therefore, cells evolve strategies for H_2_S detoxification. On the other hand, H_2_S oxidation to SO_3_^2–^ leads to sulfite toxicity, which is based on cytochrome *c*-dependent ROS production [31]. Hence the efficient oxidation of sulfite by the SOX enzyme is beneficial for mitochondrial function.

Till now, there are no available data explaining either how sulfide is detoxified in fungal mitochondria or what the mechanism of oxygen sensing is. However, the mechanism of sulfide oxidation might be similar to that in human mitochondria, since a strongly elevated SQR transcript level was observed in the sulfur-regulatory *sconB* mutant, known to have derepressed sulfate assimilation pathway and elevated sulfide production [32].

Both menadione and antimycin A promote AstA-mitochondrion shift at a low temperature (28 °C). We may suppose that, in each case, the mechanism of action is different. ROS-generating compound menadione changes the redox balance by decreasing the level of reduced glutathione [33], needed for sulfide detoxification. As a consequence, excess free sulfide triggers the SQR–SDO–rhodanese pathway [32], leading to its oxidation to S^4+/6+^derivatives (SO_3_^2−^, S_2_O_3_^2−^). However, by blocking Complex III, antimycin A leads to the activation of electron bypass by oxidizing sulfide into sulfite through the SQR–SDO–rhodanese pathway [9,10]. In both cases, AstA might then help to remove excess sulfite and thiosulfate anions by transporting them into the mitochondrial intermembrane space (Figure 6B).

Furthermore, we showed that AstA may tune ATP levels or sulfite oxidase (SOX) activity depending on the available sulfur source. In the *astA*-overexpressing strain, cysteine, as a reactive thiol compound and the source of toxic sulfide, may trigger a response of the SQR–SDO–rhodanese pathway. Oxidation of H_2_S is then the alternative source of electrons, and improves ATP synthesis. Significantly elevated ATP levels were observed when strains were grown in the presence of cysteine, while other sulfur sources remained without effect or decreased ATP amounts in the *astA*-overexpressing strain. The SOX enzyme, which is responsible for sulfite detoxification in the mitochondrial intermembrane space, also displayed significantly elevated activity in the presence of cysteine. Because cysteine contains a reactive sulfhydryl group and is the main source of sulfide in the cell, excess of derived S^2–^ serves as an additional oxidation acceptor in the respiratory chain, which correlates with the elevated amount of ATP (Figure 5B). One of the final sulfide-oxidation products, thiosulfate, is then effluxed by AstA into the mitochondrial intermembrane space, and oxidized to sulfate by the induced SOX enzyme. On the other hand, when sulfite serves as a sole sulfur source, the level of ATP remains lower than in the wild-type strain despite elevated SOX activity. This may be due to the toxic effects of cytochrome *c*-inhibition by sulfite [31], which is strongly fluxed by AstA into the mitochondrial intermembrane space (Figure 6B).

Hence, respiratory-chain tuning by AstA may be particularly beneficial for pathogenic or endophytic fungi when they activate the highly energy-consuming biosynthesis of hydrolytic enzymes, which is required for host-tissue decomposition. Steady-state production of sulfide might then occur in the mitochondrial matrix, as is the case in human mitochondria [9,10]. AstA, which is then translocated to the mitochondrial membrane in an Hsp70–Porin1-dependent manner (Figure 6A) may participate in the efflux of oxidation products sulfite and thiosulfate out to the intermembrane space (Figure 6B). Thereby, AstA might be a missing auxiliary element of the SQR–SDO–rhodanese pathway, present in the *astA*-possessing fungi. This way, AstA may modulate respiratory-chain activity and ATP synthesis, improving mitochondrial activity. On the other hand, under liquid-growth conditions or in lower temperatures, there is no recruitment of the AstA protein into the mitochondria, and ATP-independent AstA undergoes insertion mostly into the cell membrane for sulfate uptake (Figure 6C). However, more detailed elucidation of the function of AstA in the mitochondria of fungi requires further studies.

## 4. Materials and Methods

### 4.1. Strains and Media

*Aspergillus nidulans* strains from our collection, carrying standard markers [34,35] and used in this study, are listed in Table 1 with the *Escherichia coli* strain.

### 4.2. Growth Conditions

The following media (solid and liquid) were used: complete medium (CM) [36] for protoplasts or DNA isolation, minimal medium (MM), or minimal sulfur-free medium (MM-S) [37], the latter supplemented either with an inorganic sulfur source, including sulfate, sulfite, or thiosulfate (0.1 mM, 1 mM), or organic sulfur source L-methionine (0.1 and 1 mM). The minimal media were also supplemented according to the auxotrophic requirements of the employed strain or with compounds mentioned in Section 2 and Table 1. In case of growth on solid medium, strains were indirectly grown on cellophane discs at 37 °C for ATP and enzymatic assays. Liquid cultures were grown at 28 or 37 °C for 16 h in a rotary shaker (200 rpm). *Escherichia coli* was grown in the standard LB medium supplemented with antibiotics as required [38].

### 4.3. Nucleic Acid Manipulation and Plasmid Construction

Used plasmids are listed in Table 1. Standard procedures for plasmid propagation and isolation were according to published methods [38]. DNA was isolated from *A. nidulans* by the salting-out method. Frozen mycelia were disrupted by grinding in liquid nitrogen, followed by immediate suspension in a warm STEN buffer (1% SDS, 100 mM Tris pH 7.5, 50 mM EDTA pH 8, 100 mM NaCl) [38]. Polymerase chain reactions (PCR) were performed in a Bioneer Thermocycler (Bioneer, Daejeon, Korea). DNA was sequenced, and primers were synthesized by the DNA Sequencing and Oligonucleotide Synthesis Laboratory, Institute of Biochemistry and Biophysics, PAS. Sequences of the used primers are provided in Supplementary Table S2. To construct plasmid-expressing fused *N*-terminal 37 amino acid of AstA to GFP protein, the NsiI-SnaBI of 3′*astA* ORF fragment was cloned into pEGFP–N1 vector (Clontech, Mountain View, CA, USA), also bearing gentamycin selectable cassette, used further for selection of proper transformants. Plasmid kPMS11-5243 used for *astA*-GFP overexpression was previously described [3]. To disrupt the Por1 gene, AN4402 locus was PCR-amplified with primer pair U_Por_-L_Por_, cloned into pGEM-T Easy vector (Promega, Madison, WI, USA) yielding the pGEMPor1 plasmid, and the BamHI-BamHI *pyr-4* selectable cassette fragment was ligated into pGEMPor1 BglII site yielding the pGEMPor1::*pyr-4* plasmid. Southern blot analysis was performed as previously [1].

**Table 1:** List of strains and plasmids.

Designation	Genotype or Relevant Features	Reference or Source
*E. coli* strain XL1 Blue	*recA1 endA1 gyrA96 thi-1 hsdR17 supE44 relA1 lac* [F’ *proAB lacI*^q^ *Z*Δ*M15* Tn10 (Tetr)]^c^	Stratagene
*A. nidulans* strains		
W1 (WT)	*pyroA4 yA2*	[34]
W13	*pyrG89 pyroA4 yA2*	[39]
W13 [*astA*^+^] (WT [*astA*^+^])	*pyrG89* [*astA*^+^ *Ncpyr-4*^+^] *pyroA4 yA2*	[3]
WT [^37Nterm^*astA*-GFP^+^, mitoRFPff^+^]	*pyroA4 yA2* [^37Nterm^*astA*-GFP^+^::*gen*, mitoRFPff^+^]	This work
WT ΔPorin	*pyrG89* [*por1::Ncpyr-4^+^*] *pyroA4 yA2*	This work
WTΔPorin [ *astA*-GFP^+^, mitoRFPff^+^]	*pyrG89* [*por1::Ncpyr-4^+^*] *pyroA4 yA2* [*astA*-GFP^+^, mitoRFPff^+^]	This work
**Plasmids**	Description	
pBluescript KS(–)	Cloning vector, Amp^r^	Stratagene
pGEM-T Easy	Linear vector for cloning of PCR products	Promega
HELp1	Increases *A. nidulans* transformation efficiency about 200 fold	[40]
pEGFP-N1	Vector bearing GFP-encoding ORF and gentamycin selectable cassette	Clontech
pEastA^37Nterm^GFP-N1	pEGFP-N1 plasmid bearing the *astA* ORF 5′ sequence fragment encoding first 37 amino acids	This work
pXY142-mtRFPff	Bearing nucleotide sequence encoding *N. crassa* H^+^ mitochondrial ATPase leader fused to fast folding RFP-encoding sequence	Prof. Shaw gift
kPG-26B	Bearing entire *N. crassa pyr-4* in pBluescript II KS(−)	laboratory collection
kPMS11-52	BamHI-SalI insert bearing the *astA* gene in pBluescript KS(–) vector	[1]
kPMS11-5243 (*astA*-GFP)	kPMS11-524-derived *astA* gene fused with GFP-encoding gene with *N. crassa pyr-4* cassette	[3]
pGEMPor1	pGEM-T Easy bearing PCR fragment of entire AN4402 locus (porin)	This work
pGEMPor1::*pyr-4*	pGEMPor1 bearing disrupted AN4402 locus with *N. crassa pyr-4*	This work

### 4.4. Transformation of A. nidulans

The preparation of protoplasts and their transformation with plasmids were performed as described earlier [3]. The W13 strain was transformed with the pGEMPor1::*pyr-4* plasmid to uracil prototrophy, and transformants were first PCR-selected with primer pair U_Por_-L_Por_ to select the proper *Por1::pyr-4* knock-out isolates, which were further confirmed by Southern analysis (Supplementary Figure S1). Plasmid pXY142-mtRFPff bearing nucleotide sequence encoding *N. crassa* H^+^ mitochondrial ATPase leader [41] was modified by Prof. Shaw (kind gift) by fusing it with fast-folding Red Fluorescent Protein mRFPff, and was used to cotransform *A. nidulans* strains in order to visualize mitochondria under confocal microscope.

### 4.5. ATP Measurements and Sulfite Oxidase Assay

Rhodamine B-derived ATP sensor (RSL) was synthesized as described [42] in the Department of Biophysics, IBB PAS. RSL-ATP binding was verified under the microscope and in vitro using ATP for standardization. The mycelia of particular strains were collected from cellophane discs of solid growth cultures, grinded in 50 mM Tris-HCl pH 8.5, and, after centrifugation at 14,000 rpm/5 min, supernatant was used for analysis. RSL compound was used in a final concentration of 10 μM, and excited fluorescence at 520 nm was read at 583 nm. Sulfite oxidase (SOX) assay was based on a two-step method [43], with the SOX-HRP-coupled oxidation of diaminobenzidine (DAB) to a colorimetrically counted product with absorbance at 336 nm, using 1 mM sulfite as a substrate. SOX activity ratio was estimated on the basis of only absorbance. Protein concentration was estimated as described [44]. Assays were carried out in 96-wheel plates and read in Varioscan^TM^ LUX multimode microplate reader (Thermo Fisher Scientific Oy, Vantaa, Finland).

### 4.6. Confocal Microscopy

Mycelia for microscopic observations were grown in a liquid or on a solid MM-S medium supplemented with 0.1 mM methionine or 0.1 mM sulfate (derepressing conditions). Liquid cultures were grown for 18 h at 28 or 37 °C in a rotary shaker (200 rpm); on solid media, strains were cultivated for 48 h at 28 or 37 °C on glass slides covered up with thin solid medium in Petri plates. For visualization of the mitochondria, Rhodamine 123 was also used. For each observation, more than 50 hyphae were scored. Presented data are representative of at least five independent experiments with essentially identical results. Colocalization analysis was performed by comparing the Pearson’s coefficient ImageJ intensity-correlation-analysis plugin, which determines the relative intensities of GFP- and RFP-induced fluorescence in the same groups of pixels delimited by the selected region of interest (ROI). Mycelial samples were examined under an Olympus Fluoview FV10i confocal microscope (Olympus, Tokyo, Japan) with 600× magnification, and digital data were further analyzed with Imaris ×64 7.7.2. software.

### 4.7. Bioinformatic Analysis

The *A. nidulans* AstA protein (Acc.no. ABA_28286) was queried against NCBI’s GenBank nonredundant protein database (nr) by BLASTP and genomic raw data by BLAST [45]. Putative subcellular localization of AstA or proteins encoded by the genes encompassing *astA* locus were verified by freeware PSORT and SignalP algorithms [46,47].

## Supplementary Materials

Supplementary materials can be found at https://www.mdpi.com/1422-0067/21/20/7727/s1.

## Abbreviations

SOX	sulfite oxidase
GSH	glutathione
GS-SH	persulfide glutathione
RSL	rhodamine-based spirolactam
