# Supplementary figures and images for: Internalization of the Aspergillus nidulans AstA Transporter into Mitochondria Depends on Growth Conditions, and Affects ATP Levels and Sulfite Oxidase Activity
Source: Int J Mol Sci. 2020 Oct 19;21(20):7727. doi: 10.3390/ijms21207727 (PMC7589619; doi:10.3390/ijms21207727)

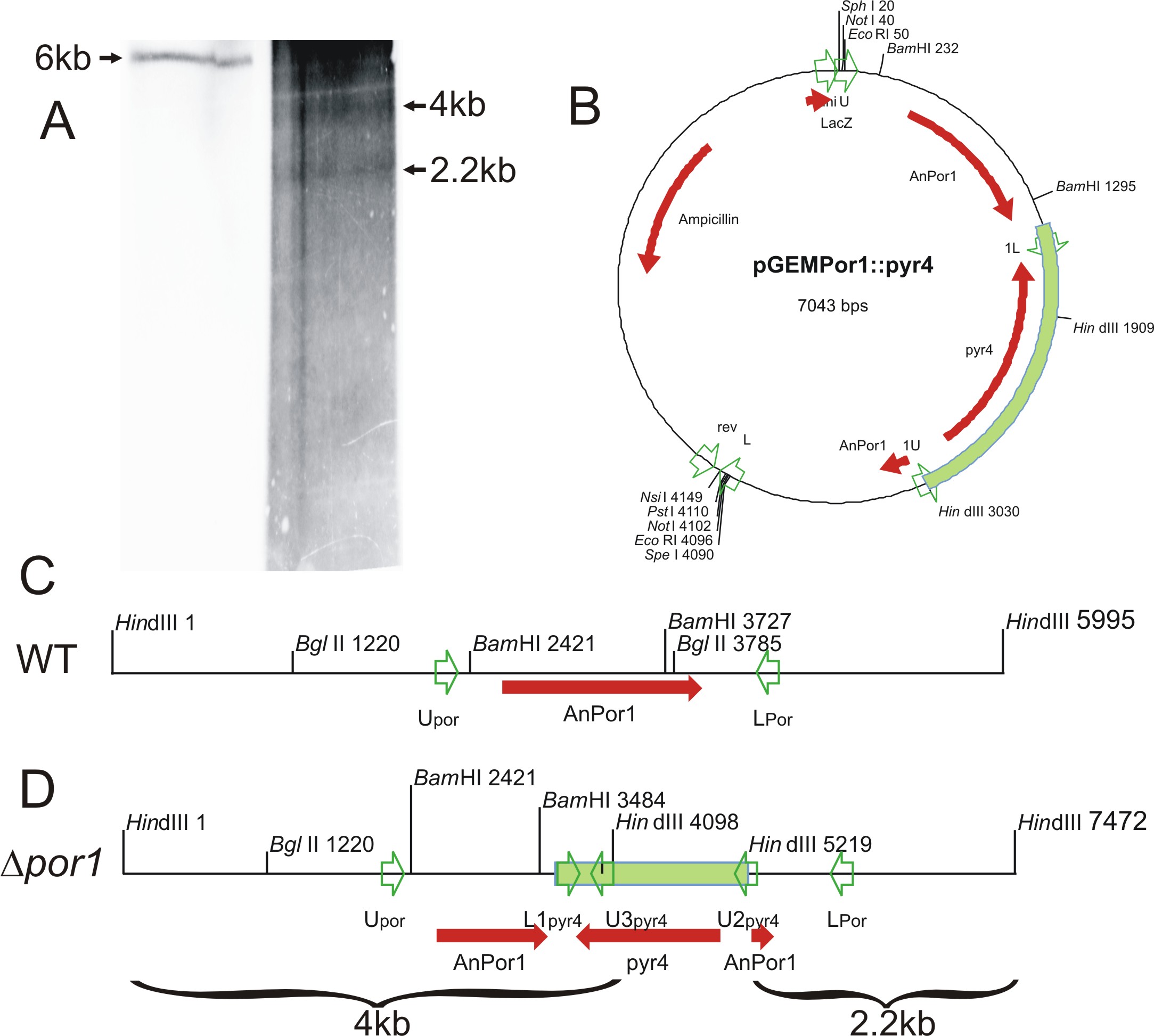

Supplement: Supplementary file 1 [file ijms-21-07727-s001.zip › Suppl Fig S1.JPG]
