# Supplementary material for: Internalization of the Aspergillus nidulans AstA Transporter into Mitochondria Depends on Growth Conditions, and Affects ATP Levels and Sulfite Oxidase Activity
Source: Int J Mol Sci. 2020 Oct 19;21(20):7727. doi: 10.3390/ijms21207727 (PMC7589619; doi:10.3390/ijms21207727)
Supplement: Supplementary file 1 [file ijms-21-07727-s001.zip › Suppl Table S2.doc]

| name | sequence (5’→3’) | description | product length [bp]/annealing temp. [°C] |
| --- | --- | --- | --- |
| U2pyr4 | TAGACTTCAACAACCCCACCA | cloning of *N. crassa* *pyr-4* selectable cassette and selection *por::pyr-4* mutants | 1481/55 |
| L1pyr4 | TATGTAGACGCTCATCCCAGT |  | 1490/60(+UPor) |
| U3pyr4 | ATTTGTAAGGCACACGGGCAAGG |  |  |
| UPor | TTCAGCGGTGCCTCGTCAAATCCTC | forward primer for cloning and selection of AN4402 locus (porin). | 2551/60 |
| LPor | GTCACGGCGCATCATATTTCGGGTC | reverse primer for cloning and selection of AN4402 locus (porin). |  |
| astA UATG | ATGGCCGACTACAAATCCACCAATC | forward primer for selection and sequencing of the pEastA**^37^**^Nterm^GFP-N1 plasmid | 2085/60 |
| AstA LGFP | CGCCGCTTGACTAGAGTCCGGACTTGTA | reverse primer for selection of astA**^37^**^Nterm^-GFP |  |

**Table S2.** Oligonucleotides used as primers for PCR amplification and sequencing.
